# Investigation of the pan-cancer property of SDC1 and its expression pattern affected patients’ overall survival for breast cancer

**DOI:** 10.1007/s12672-025-04139-x

**Published:** 2025-12-09

**Authors:** Yao Song, MeiLing Lu, Qifeng Luo, Chunhua Lu, Xiaofeng Xu, Shunjie Yuan, Bin Xu, Hua Huang, Qing Lin

**Affiliations:** 1 Department of Radiation Oncology, Shanghai Tenth People’s Hospital of Tongji University, Shanghai, 200072 China; 2 Department of Central Laboratory, Shanghai Tenth People’s Hospital of Tongji University, Shanghai, 200072 China; 3 Department of General Surgery, Shanghai Tenth People’s Hospital, Tongji University School of Medicine, Shanghai, 200072 People’s Republic of China; 4 Department of Radiation Oncology, Lu’an Hospital Affiliated to Anhui University of Chinese Medicine, Lu’an, China; 5 Department of Pathology, Affiliated Hospital of Nantong University, Nantong, 226300 Jiangsu China

**Keywords:** Pan-cancer, SDC1, Breast cancer, Survival

## Abstract

Syndecan-1 (SDC1) is a transmembrane heparan sulfate proteoglycan that functions as a receptor in the extracellular matrix and plays a role in intercellular communication, cell proliferation, angiogenesis and metastasis. However, the effect of SDC1 expression on different cancers is controversial. In this study, we used The Cancer Genome Atlas (TCGA) dataset indicating that SDC1 was significantly upregulated in a wide range of cancers compared with normal tissue but was downregulated in kidney renal clear cell carcinoma (KIRC), kidney chromophobe (KICH), Kidney papillary cell carcinoma (KIRP) and Pheochromocytoma and Paraganglioma (PCPG). The expression of SDC1 was significantly associated with breast cancer subtype and this gene was found to be associated with overall survival (OS) in most analyzed cancers. From mechanistic perspective, SDC1 expression levels were correlated with DNA methylation, immune cell infiltration and tumor cell stemness in multiple cancer types based on TCGA dataset. We further investigated and validated these findings in breast cancer, SDC1 expression was significantly correlated with stem cell markers which were mainly expressed in tumor cells and stromal cells but not immune cells in the Tumor Immune Single-cell Hub 2 (TISCH2) dataset. In vivo validation, 708 breast tumors tissue microarray (TMA) analysis indicated that SDC1 protein expression in tumor cells was linked to overall survival in breast cancer patients (HR:1.54 (1.01, 2.35), P = 0.04), after adjusting for factors such as age, menopausal status, tumor characteristics, nodal involvement, TNM stage, vascular invasion, chemotherapy, and radiotherapy. In subtype analysis, SDC1 expression in tumor cells was associated with OS in the luminal subtype (HR: 1.82 (1.02, 3.25), P = 0.04), but not in human epidermal growth factor receptor 2 (HER2)-positive or triple-negative subtypes. In vitro validation, reducing SDC1 expression inhibited cell proliferation and suppressed cancer stemness biomarker expression in breast cancer cell lines. This study indicated that SDC1 expression exhibits varying impacts in pan-cancers, however, the higher SDC1 expression in breast tumor cells, but not in stromal cells, correlated with increased mortality, particularly in luminal molecular subtype, which might be a potential prognostic marker and therapeutic target for breast cancer therapy, especially in Luminal subtype breast cancer.

**Supplementary Information:**

The online version contains supplementary material available at 10.1007/s12672-025-04139-x.

## Introduction

SDC1 is a heparin cell surface proteoglycan that acts as a co-receptor for growth factors and chemokines, and it is significantly associated with tumor aggressiveness and clinical outcomes [1, 2]. The role for SDC1 as the different role in the previous research suggested that SDC1 could serve as a prognostic biomarker [3]. However, its clinical impact and prognostic significance in different cancer was debated, likely due to variations in expression levels across different tumor tissues and cancer type [4–7].

In colon cancer, the stromal cell expressed SDC1 was associated with good prognosis [8]. In pancreatic cancer, SDC1 as a critical mediator of micropinocytosis to mediated the tumor growth [9]. In breast cancer, the SDC1 expressed in different partial and different subtype associated with different affection [4, 10–12], however, the role of SDC1 in pan-cancer has been less studied.

In this study, we investigated SDC1 expression, OS and mechanism in pan-cancer. We then focused on breast cancer, checking SDC1 expression by immunohistochemical staining using primary breast cancer samples from Shanghai Tenth Hospital. We examined the relationship between different pattern of SDC1 expression and OS and event-free survival (EFS) in established tumors. Furthermore, we explored the role of SDC1 in breast cancer progression by knocking down its expression in breast cancer cells, which was validated in vitro and vivo.

## Material and methods

### Expression and prognostic analysis

Data from the TCGA dataset was used to compare SDC1 mRNA levels between tumors and normal tissues across 33 cancer types. The RNA expression data from TCGA were obtained directly from the TCGA database (https://portal.gdc.cancer.gov). The R package‘ggplot2’ was used for analyses of differential gene expression. Boxplots were used to present differences in expression levels across cancer types. SDC1 protein levels were compared between tumors and normal tissues with Clinical Proteomic Tumor Analysis Consortium data derived from the UALCAN portal (https://ualcan.path.uab.edu/, accessed on 12 March, 2025).

Univariate Cox regression analyses, conducted with the R packages ‘survival’ and ‘forestplot’, were used to evaluate the prognostic relevance of SDC1 expression for OS, disease-specific survival (DSS) and progression-free interval (PFI). The cohort was stratified into high and low expression groups based on the median level of SDC1 mRNA expression, with the lower 50% defined as the low-expression group and the upper 50% as the high-expression group.

### Genomic alteration, mutational burden, methylation analyses, immune infiltration analysis, single cell analysis and tumor stemness

Pan-cancer analyses of the frequencies of genomic mutations, amplifications, and deep deletions were conducted using the cBioPortal Cancer Type Summary module (www.cbioportal.org, accessed on 1 March, 2025). Processed Single-Nucleotide Variant (SNV) data and methylation analysis data obtained from Gene set cancer analysis (GSCA) dataset (https://guolab.wchscu.cn/GSCA, accessed on 1 March, 2025). Sangerbox platform was used to examine the correlation between SDC1 expression and tumor stemness (http://sangerbox.com/home.html, accessed on 1 March, 2025). Single cell analysis of SDC1 mRNA expression was checked using Tumor Immune Single-cell Hub 2 (TISCH2) dataset (http://tisch.comp-genomics.org/home/, accessed on 12 October, 2025).

### Functional enrichment analysis

To explore downstream pathway alterations caused by enhanced expression of SDC1, we identified DEGs between SDC1-high and SDC1-low breast cancer samples based on TCGA data using the R packages“DESeq2” and “ggplot2”, DEGs were defined using a threshold of |log2FoldChange|> 1 and an adjusted p-value < 0.05. To further clarify the potential mechanisms of SDC1 in breast cancer progression, GO and KEGG enrichment, GSEA was performed to predict the functions and pathways of the SDC1-related DEGs using the R package “clusterProfiler” “ggplot2”. The relationship between SDC1 and relevant pathway in breast cancer was conducted using the GSCA dataset (https://guolab.wchscu.cn/GSCA, accessed on 1 March, 2025). Additionally, the relationship between SDC1 and stem cell markers was analyzed with GEPIA2 (http://gepia.cancer-pku.cn/detail.php, accessed on 1 March, 2025).

### Histopathological analysis and clinical information

We collected medical and pathological data from breast cancer patients who underwent surgery between 2013 and 2018. The data included tumor size, lymph node involvement, lymphovascular invasion, chemotherapy, and radiotherapy details. The patients were assessed based on histological type, grade, and the status of estrogen receptors (ER), progesterone receptors (PR), and HER2. Patients were categorized into three molecular types according to their ER, PR, and HER2 status: Luminal-like (ER positive, with either PR and HER2 positive or negative), HER2 positive (ER and PR negative, HER2 positive), and triple negative (ER, PR, and HER2 negative). Additionally, breast cancer staging was determined according to the 8th AJCC criteria, which considers tumor size, lymph node involvement, metastasis, and tumor grade [13].

### SDC1 and CD24 expression in TMA

Breast tumors were operated at the Tenth Hospital of Tongji University, China, between 2013 and 2018, with a total of 708 breast tumor samples included in the TMAs. Each specimen contained both tumor and tumor stroma. Tissue cores were extracted from formalin-fixed paraffin-embedded (FFPE) blocks, and breast tumor diagnoses were confirmed by a pathologist following Hematoxylin and Eosin staining. TMA analysis (conducted by Shanghai Outdo Biotech, Shanghai, China) involved 2-mm tissue cores taken from two areas of the tumor in each patient (the invasive margin and tumor bulk). We examined various pathological features, including tumor size, lymph node status, tumor grade, hormone receptor (HR) status, and HER2 status. The median follow-up duration was 6.64 years (ranging from 6.42 to 6.91 years). Ethical approval for this research was granted by The Human Research Ethics Committee of Tenth Hospital of Tongji University, and all patients provided informed consent.

The staining procedures for CD24 and SDC1 were performed as previously described [6]. In brief, antigen retrieval was achieved through microwave pre-treatment in EDTA buffer (pH 9.0) for 20 min, followed by the removal of endogenous peroxidase with 3% H_2_O_2_ and a 20-min blocking step with avidin. CD24 (ab31622, Abcam, U.K.) and SDC1 (ab7280, Abcam, U.K.) were then incubated with secondary antibodies at room temperature for 30 min. Tissue sections were treated with 3,3-diaminobenzidine for 10 min, then counterstained, dehydrated, and mounted.

The expression levels of SDC1 in tumor and stromal cells were independently evaluated by two pathologists using the immunoreactive score (IRS) [14]. The IRS was calculated by multiplying the scores for staining intensity and staining extent. Staining intensity was scored as follows: 0 (negative), 1 (weak), 2 (medium), and 3 (strong). Staing extent as follows: 0(0%), 1(< 10%), 2 (10–50%), 3(51–80%) and 4(80–100%),The result was graded as follows:0(score 0–1), 1 (score 2–3), 2 (score 4–8) or 3 (score 9–12). Here, grade 0 and1 represent low expression; while grade 2 and 3 represent high expression, which were considered positive for both tumor and stromal cells. The expression of CD24 and SDC1 in tumor tissue was assessed as previously described [5].

### Cell culture and transfection of tumor cell

The human breast cancer cell lines MDA-MB-231 and MCF-7 were obtained from the Chinese Academy of Medical Sciences (ATCC ®HTB-26™, Beijing, China). Cells were cultured in DMEM medium supplemented with 10% fetal bovine serum at 37 °C in a 5% CO2 incubator. For gene knockdown, MDA-MB-231 and MCF-7 cells were seeded in 6-well plates and incubated for 24 h. They were then transfected with either SDC1-targeting siRNA or a non-targeting control siRNA (siCon) using Lipofectamine 8000 reagent (Beyotime, Shanghai, China). The siRNA sequences used were as follows: siSDC1: sense: GGACUUCACCUUUGAAACC; antisense: GGUUUCAAAGGUGAAGUCC; siSDC1-2: sense: GGAGGAAUUCUAUGCCUGA; antisense: UGUUUCUUUCAU UGCAUUU. Follwing transfection, cells were continued to be cultured under the same conditions.

### Cell Counting Kit-8 (CCK-8) assay

The cells were plated in 96-well plates at a density of 2000 cells per well and cultured for various time intervals. Following this, 10μL of CCK-8 reagent (share-bio, Shanghai, China) was added to 100 μL of the culture medium. After a 2-h incubation at 37 °C, the absorbance was measured at 450 nm using an Microwell Plate Spectrophotometer (BioTek, Winooski, VT).

### Quantitative real-time PCR (RT-qPCR)

Total RNA was isolated and reverse transcribed to cDNA as previously described [15]. Reverse transcription into cDNA was done using PrimeScript ™ RT Master Mix (Perfect Real Time) Kit (RR036A, TAKARA, Kusatsu, Japan). RT-qPCR was performed by Fast SYBR^®^ Green Master Mix (4,385,612, TermoFisher Scientific) on QuantStudio™ 7 Flex Real-Time PCR System (4,485,701, TermoFisher Scientific). The expression levels were normalized to GAPDH. The primer sequences for SDC1 and GAPDH as follow: GAPDH-F 3ʹGCCAAAAGGGTCATCATCTC5ʹ GAPDH-R 3ʹTGAGTCCTTCCACGATACCA5ʹ; SDC1-F 3ʹCTGGTGGGTTTCATGCTGTA5ʹ; SDC1-R 3ʹCCTGTTTGGTGGGCTTCT5ʹ.

### Westernblot analysis

Proteins were extracted from tumor cell lines by using RIPA lysis buffer (Beyotime, Shanghai, China). The protein concentration was measured using a BCA Assay Kit (Pierce Biotechnology, Rockford, USA). Proteins were separated by sodium dodecyl sulfate–polyacrylamide gel electrophoresis and electro blotted onto Nitrocellulose membranes (Millipore, Bedford, MA, USA). The membranes were blocked with 5% skim milk for one hours at room temperature and then incubated overnight at 4 °C with the primary antibodies. The primary antibodies used included anti-CD133 (Cell Signaling Company, Danvers, MA, USA), anti-SOX2 (Cell Signaling Company, Danvers, MA, USA), anti-CD44 (Cell Signaling Company, Danvers, MA, USA), anti-NANOG (Cell Signaling Company, Danvers, MA, USA) and anti-GAPDH (Cell Signaling Company, Danvers, MA, USA) were used to probe the target proteins. IRD ye-labeled secondary antibodies (Li-COR Biosciences, Lincoln,NE, USA) was used as a secondary antibody and incubated with the membrane for two hours at room temperature. The protein bands were visualized using an electrochemiluminescence reagent as previously described [16].

### Statistics

Continuous variables were expressed as the mean and standard deviation (SD) or median and interquartile range (IQR), and were compared using the Wilcoxon rank-sum test or Student’s independent t-test. Categorical variables were expressed as frequency counts with percentages, and were compared using the chi-square test or Fisher’s exact test.

Univariable and multivariable analyses with the Cox proportional-hazards regression model were used to estimate the effects of prognostic factors on survival, with or without adjustment for age, menstruation, tumor, nodal, TNM stage, radiotherapy and chemotherapy. The covariates that were considered clinically relevant or that showed a univariate relationship with outcome were entered into multivariable Cox proportional-hazards regression models. Variables for inclusion were carefully chosen to ensure parsimony of the final models. Hazard ratios (HRs) and their corresponding 95% confidence intervals (CIs) were derived from Cox models. The proportionality assumption of the Cox models was tested with log–log Survival curves and Schoenfeld residuals, and were valid for all the analyses. Kaplan–Meier curves obtained with the log-rank test were plotted to demonstrate the differences of survival between groups. All analyses were performed using the open-source statistical software R version 4.2.0 (R Foundation). A threshold of p < 0.05 (two-sided) was considered statistically significant.

## Results

### Pan-cancer analyses of SDC1 expression and prognostic relevance

**Fig. 1:**
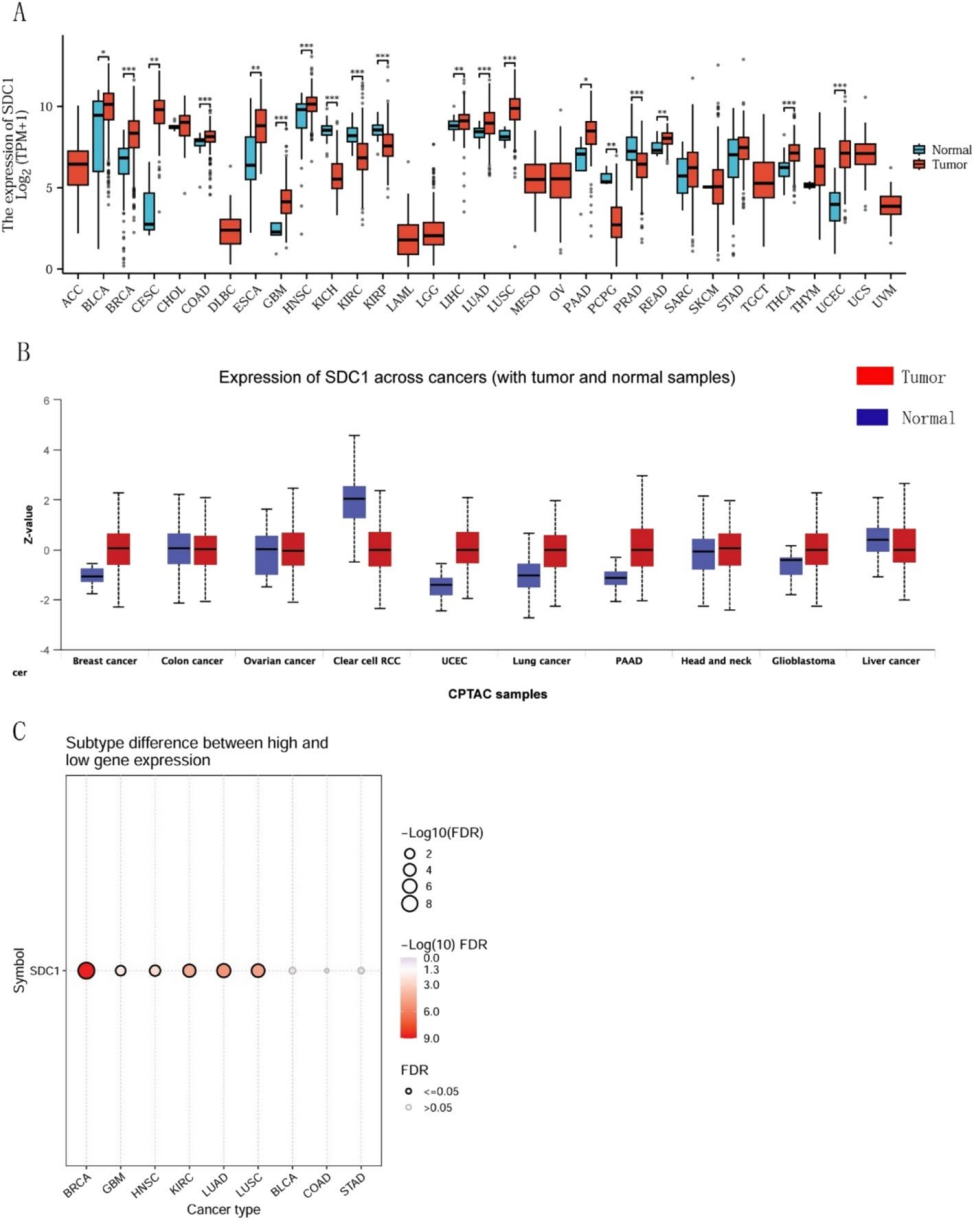
Pan-cancer analysis of SDC1 expression. **A** The transcriptional expression of SDC1 for pan-cancer in TCGA dataset. **B** UALCAN analyses the SDC1protein expression for pan-cancer in tumor and normal tissue. **C** The subtype difference between SDC1 high and low expression

A systematic pan-cancer analysis of SDC1 mRNA expression in normal and tumor tissues was conducted using the TCGA dataset.19 of 33 cancers had significantly different expression was observed. At the protein level, data from the UALCAN database revealed that SDC1 was significantly upregulated in breast cancer, uterine corpus endometrial carcinoma (UCEC), lung cancer, pancreatic adenocarcinoma (PAAD), and glioblastoma (GBM), but downregulated in clear cell renal cell carcinoma (KIRC) and liver cancer (LIHC). Subtype analysis demonstrated that significantly difference between high and low SDC1 expressed in Breast invasive carcinoma (BRCA), GBM, head and neck squamous cell carcinoma (HNSC), KIRC, Lung adenocarcinoma (LUAD) and Lung squamous cell carcinoma (LUSC) (Fig. 1A–C).

**Fig. 2:**
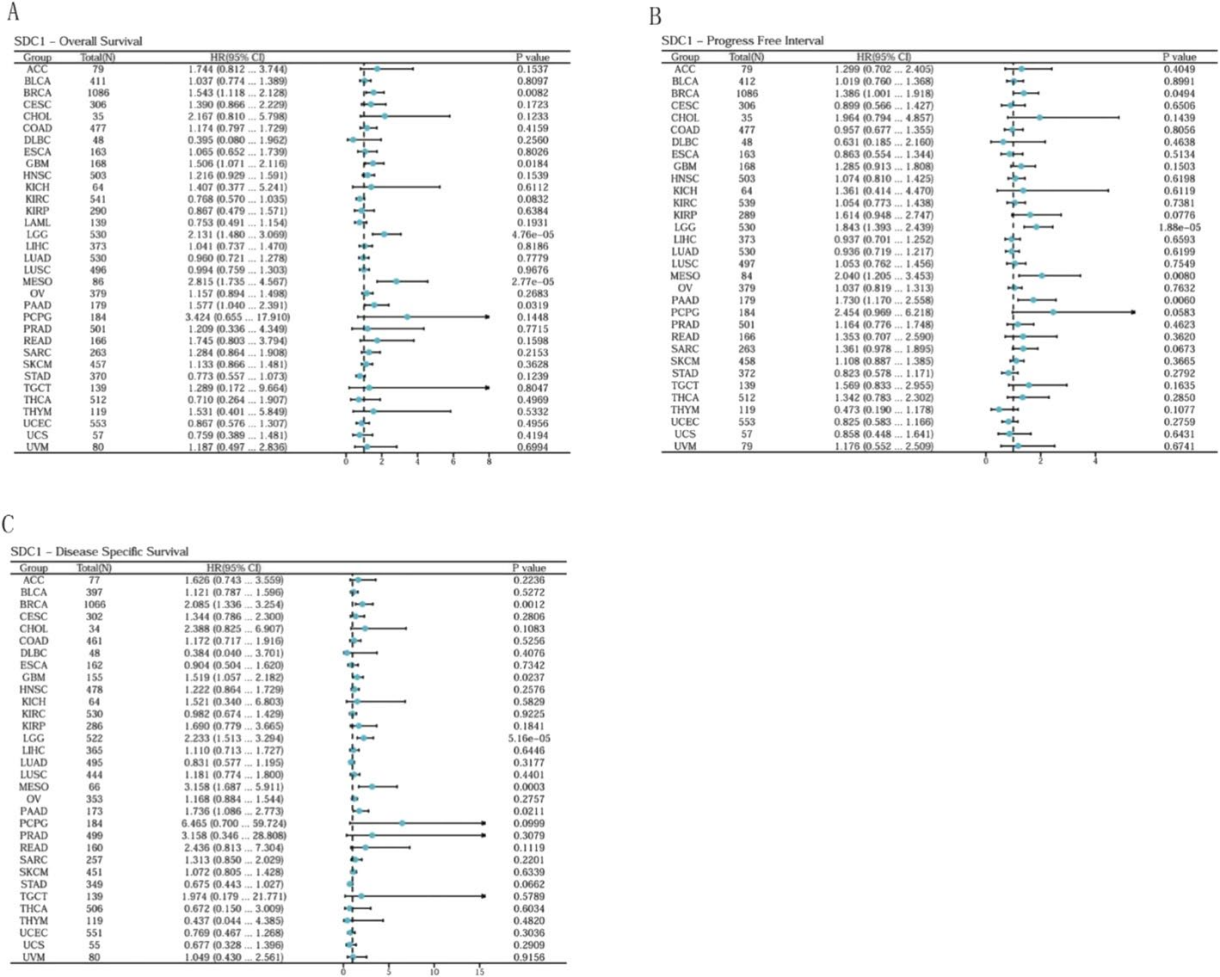
Pan-cancer analysis of SDC1 prognosis relevance. **A** Forest plots were used for pan-cancer analyses of SDC1 and OS (**A**), PFI (**B**), DSS (**C**)

Univariate Cox regression analyses indicated that high SDC1 expression was significantly associated with poor OS in BRCA, GBM, LGG, Mesothelioma (MESO) and PAAD. Similarly, SDC1 was also a risk factor associated with worse DSS in BRCA, GBM, LGG, MESO and PAAD. With respect to PFI, SDC1 as a risk factor in BRCA, LGG and PAAD (Fig. 2A–C).

### Analyses of SDC1 genomic alteration and immune activity in pan-cancer

**Fig. 3:**
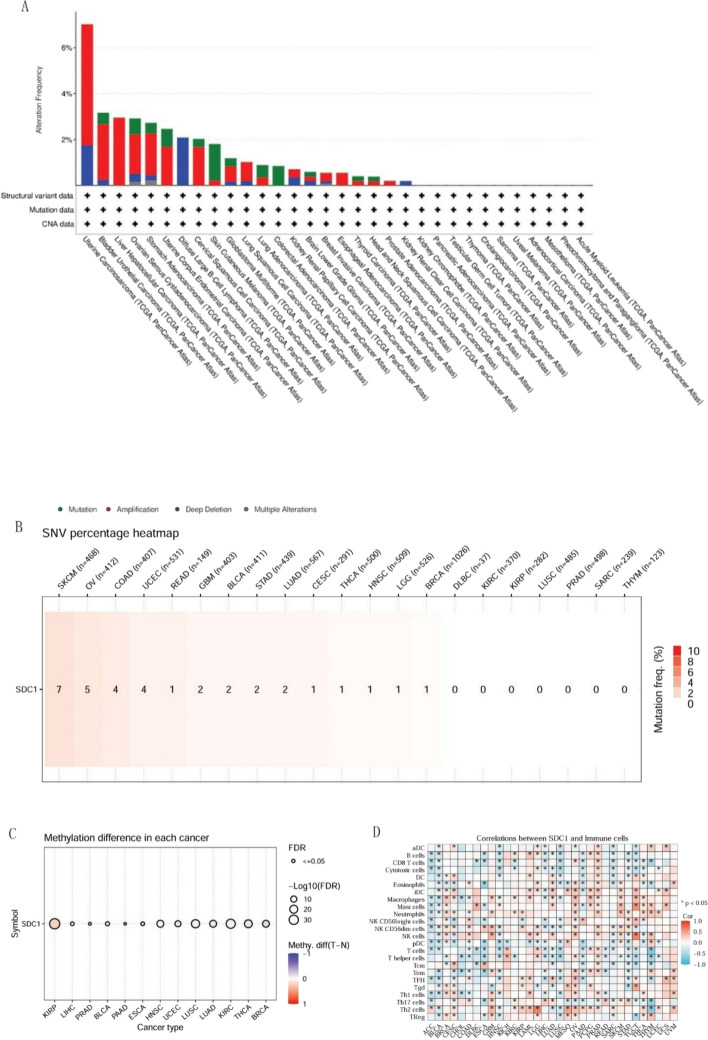
SDC1 expression associated with genomic alteration and immune activity in pan-cancer. **A** SDC1 mutations, amplifications, and deep deletions in pan-cancer conducted in TCGA database. **B** The SDC1 heatmap for SNV percentage in pan-cancer. **C** The SDC1 methylation difference in pan-cancer. **D** Correlations between SDC1 expression and immunity cells infiltration in pan-cancer

To assess potential genomic alterations of SDC1 across cancers, we performed a pan-cancer analysis of its single-nucleotide variants (SNVs) and methylation status. SDC1 amplification was most frequently observed in UC, BUC, LHC, OS and UCEC; whereas deep deletion was most prevalent in UC and DLBCL. SNV analysis revealed a high mutation rate of SDC1 in pan-cancer, Skin Cutaneous Melanoma (SKCM), Ovarian serous cystadenocarcinoma (OV), Colon adenocarcinoma (COAD) and UCEC. Methylation analysis demonstrated that the expression of methylated SDC1 expression was much higher in tumor tissue than in normal tissue in KIRP. In immune correlation analysis, SDC1 was positively associated with different immune cell infiltration in TGCT, BRCA, LGG and OV (Fig. 3A–D).

### Tumor stemness and SDC1 expression

**Fig. 4:**
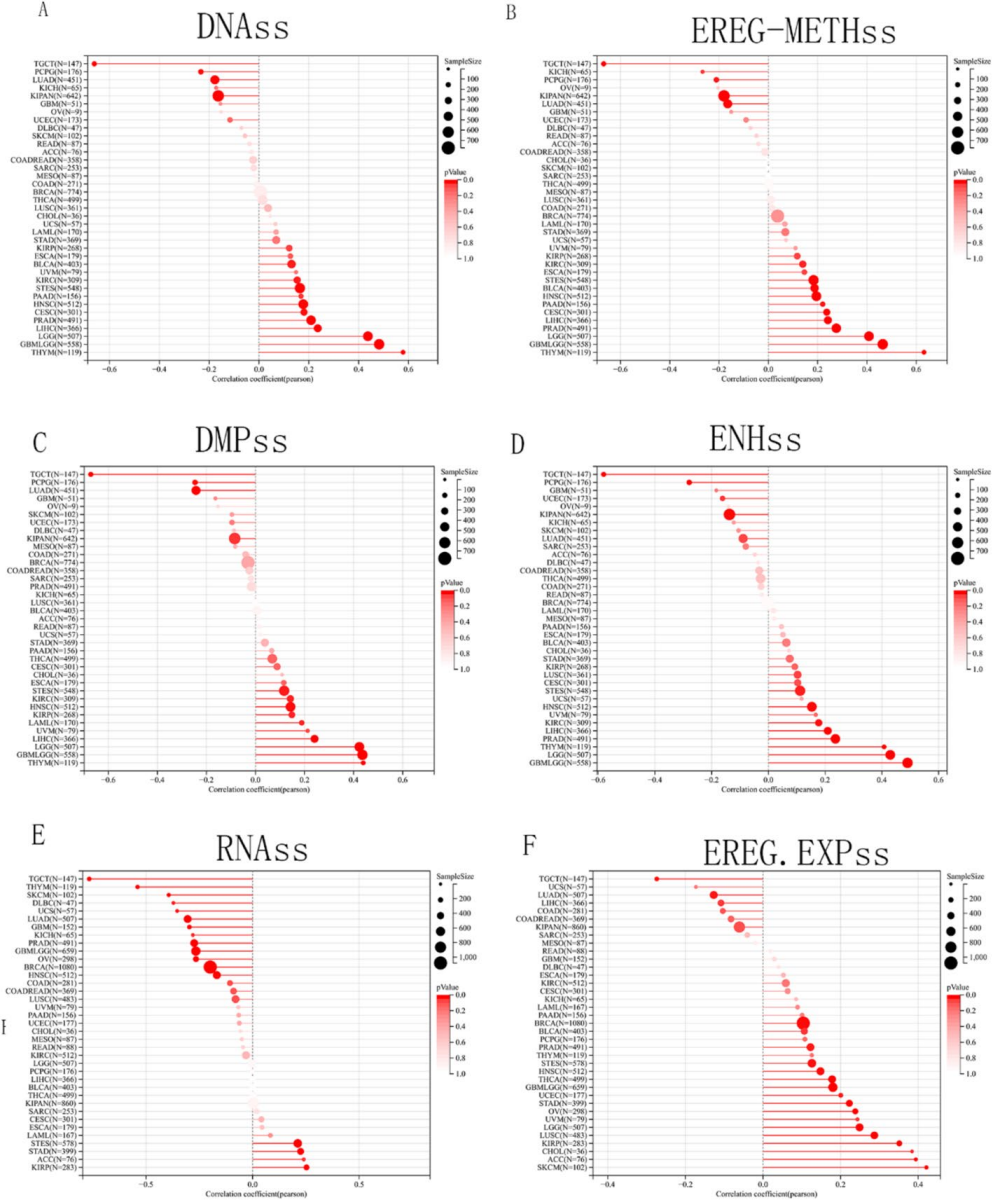
The role of SDC1 expression in cancer stemness. The correlations between SDC1 expression and **A** DNAss, **B** EREG.METHss, **C** DMPss, **D** ENHss, **E** RNAss, **F** EREG.EXPss in pan-cancer. DMPss, differentially methylated probes-based; DNAss, DNA methylation-based; ENHss, enhancer elements/DNA methylation-based; EREG.EXPss, epigenetically regulated RNA expression-based; EREG-METHss, epigenetically regulated DNA methylation-based; RNAss, RNA expression-based

Using the Sangerbox platform, we investigated the relationship between SDC1 expression and tumor stemness scores across six dimensions. The analysis revealed that SDC1 was positively correlated with the scores for DNA, EREG-methss, DMPss, ENHss, RNAss and EREG in STES, THTM. In contrast, SDC1 expression was negatively correlated with the scores for DNA, EREG-methss, DMPss, ENHss, RNAss and EREG in Testicular Germ Cell Tumors (TGCT), LUAD. Notably, in breast cancer, SDC1 expression was negatively correlated with the scores for RNAss and positively correlated with EREG (Fig. 4A–F).

### The Enrichment Function for SDC1 analysis in breast cancer

**Fig. 5:**
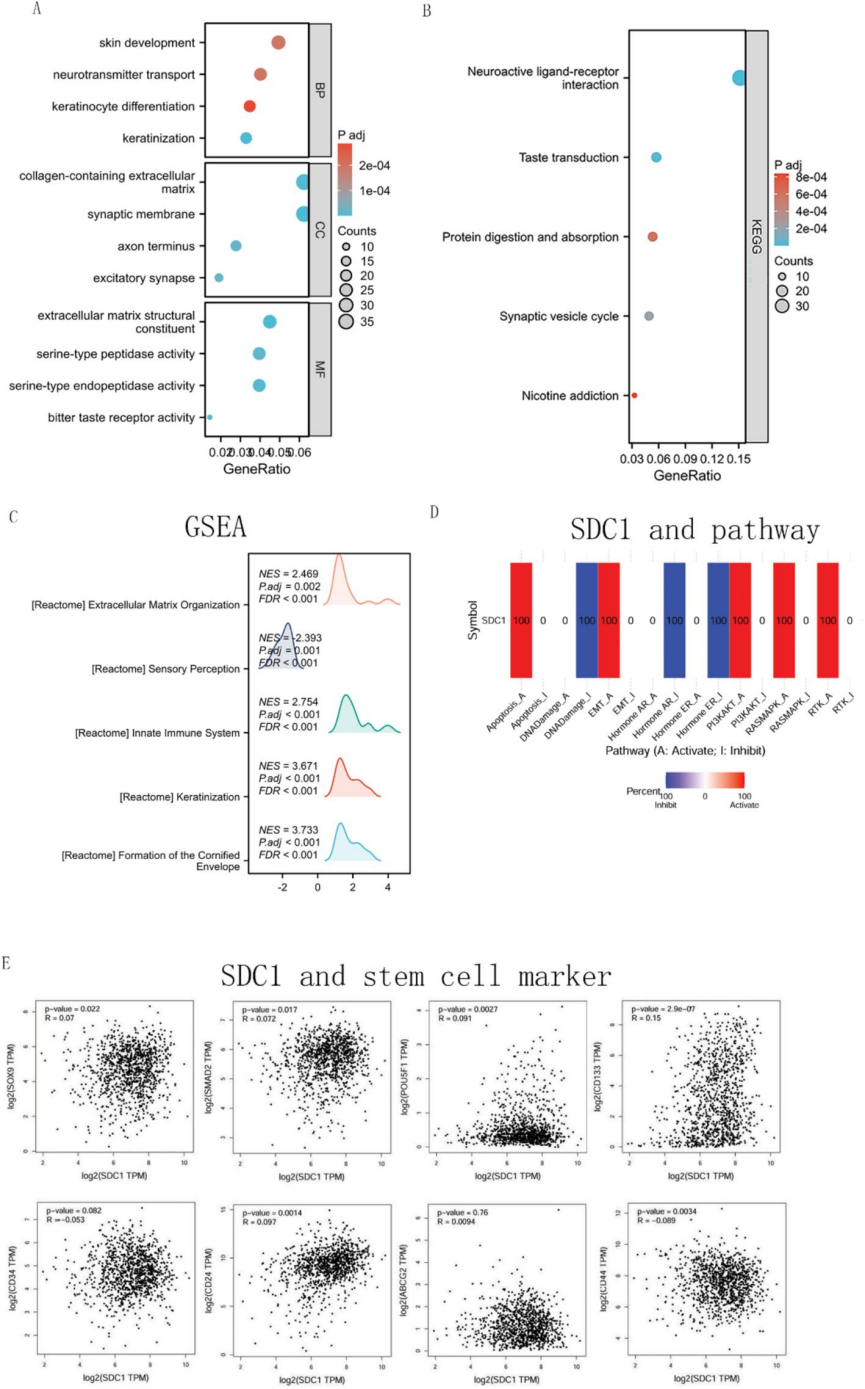
Enrichment function for SDC1 in breast cancer. **A** GO analysis of the DEGs based on the SDC1 expression in breast cancer. **B** KEGG analysis of the DEGs based on the SDC1 expression in breast cancer. **C** GSEA analysis of the DEGs based on the SDC1 expression in breast cancer. **D** SDC1 and associated pathway in high and low SDC1 expressions. **E** The correlation of SDC1 expression and stem cell markers expression in breast cancer tissue in TCGA data-set

SDC1 co-expression networks were studied using TCGA database to verify the potential function of SDC1 in tumor tissue. A total of 221 genes were significantly positively correlated with SDC1, while 401 genes were significantly negatively correlated. The most enriched Biological Process (BP), Cellular Component (CC) and molecular function (MF) were the Keratinocyte differentiation, collagen-containing extracellular matrix and extracellular matrix structural constituent; The KEGG pathway was neuroactive ligand-receptor interaction. Key genes correlated with SDC1 included SDC2, MYC, and RAF1. Furthermore, SDC1 was associated with multiple oncogenic processes, apoptosis activity, DNA damage inhibiton, EMT activity, PI3K/AKT activity, RAS/MAPK activity and ER inhibition. In the TCGA breast cancer dataset, SDC1 expression was notably correlated with the stem cell biomarkers CD133, CD44, CD24, POU5F1, SMAD2, and NANOG at the transcriptional level (Fig. 5A–E).

### Expression pattern of SDC1 expression in breast cancer

**Fig. 6:**
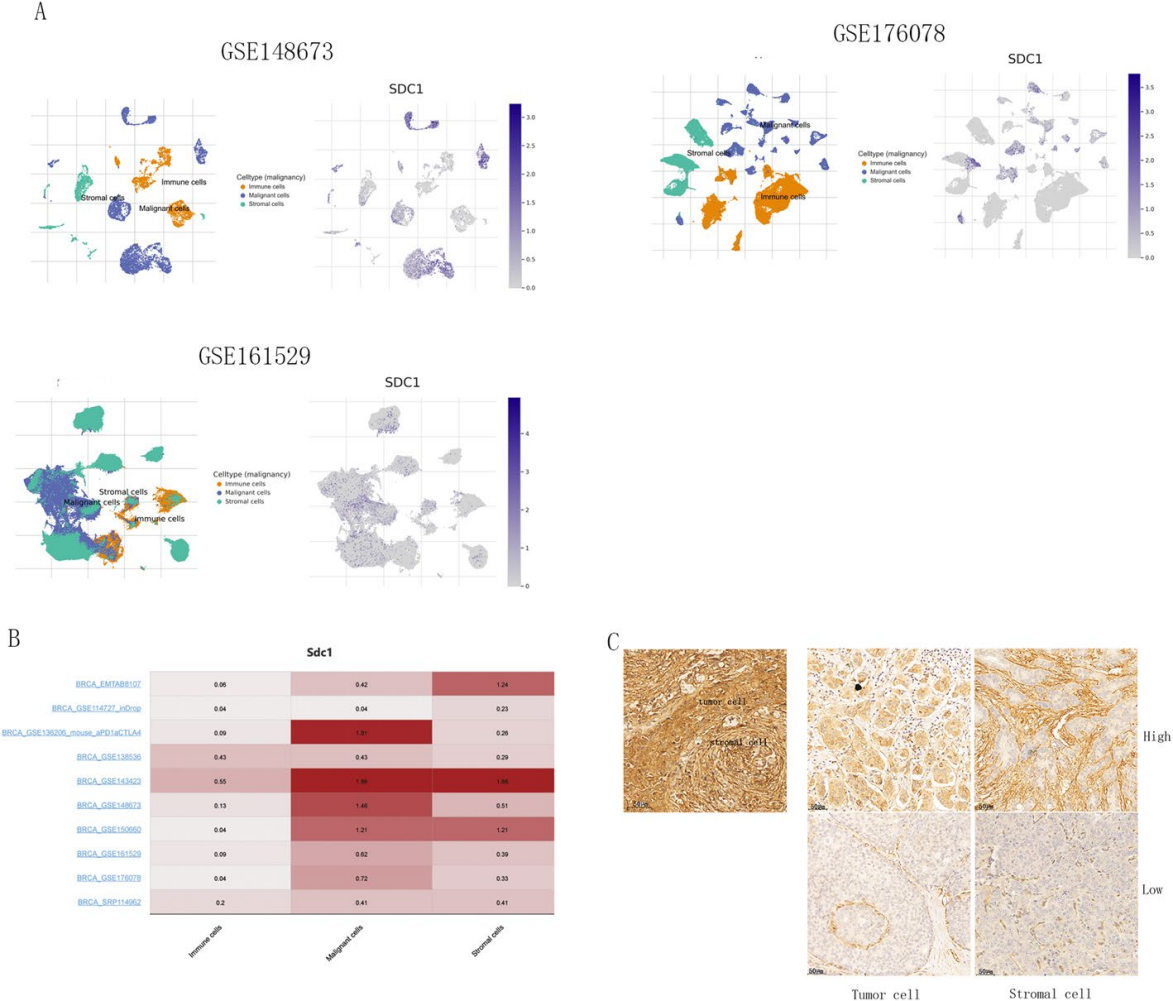
Expression pattern of SDC1 expression. **A** TISCH checking the SDC1-expressing cells in breast cancer in GSE176078, GSE161529 and GSE148673; **B** The SDC1 expression level in breast cancer different cells for different datasets. **C** The high and low expression of SDC1 in tumor cell and stromal cell

Analysis via the TISCH database indicated that SDC1 in breast cancer is primarily expressed in stromal cell and tumor cells, but not in immunity cells. IHC detection confirmed the presence of SDC1 in nearly all carcinoma areas, with prominent localization within the tumor cell cytoplasm. The staining patterns and intensity are depicted in (Fig. 6A–C).

### The validation of SDC1 expression and survival of breast cancer patients on protein level in vivo.

This research involved 708 breast cancer patients, revealing that clinical factors such as menstrual status, tumor size, nodal involvement, TNM stage, vascular invasion, and treatment with radiotherapy and chemotherapy was significantly associated with event-free survival (EFS) and overall survival (OS), as detailed in Tables 1 and S1.

**Table 1:** The clinical characters of 708 breast cancer patients

	Total (n = 708)	Death (n = 603)	Living (n = 105)	P-value
Age(average ± SD)	57.95 ± 12.39	56.94 ± 11.84	63.72 ± 13.88	< 0.001
Age				0.002
< 50	172 (24.29%)	159 (26.37%)	13 (12.38%)	
≥ 50	536 (75.71%)	444 (73.63%)	92 (87.62%)	
Menstruation				0.001
Peri- and Pre-	217 (30.65%)	200 (33.17%)	17 (16.19%)	
Post-	487 (68.79%)	399 (66.17%)	88 (83.81%)	
unknown	4 (0.56%)	4 (0.66%)	0 (0.00%)	
Tumor				< 0.001
≤ 2 cm	368 (51.98%)	332 (55.06%)	36 (34.29%)	
2-5 cm	310 (43.79%)	250 (41.46%)	60 (57.14%)	
> 5 cm	29 (4.10%)	20 (3.32%)	9 (8.57%)	
unknown	1 (0.14%)	1 (0.17%)	0 (0.00%)	
Nodal				< 0.001
0	405 (57.20%)	363 (60.20%)	42 (40.00%)	
1–3	160 (22.60%)	141 (23.38%)	19 (18.10%)	
4–9	82 (11.58%)	64 (10.61%)	18 (17.14%)	
≥ 9	61 (8.62%)	35 (5.80%)	26 (24.76%)	
TNM stage				< 0.001
I	248 (35.03%)	227 (37.65%)	21 (20.00%)	
IIA	269 (37.99%)	239 (39.64%)	30 (28.57%)	
IIB-IIIA	122 (17.23%)	95 (15.75%)	27 (25.71%)	
IIIB-IIIC	64 (9.04%)	37 (6.14%)	27 (25.71%)	
unknown	5 (0.71%)	5 (0.83%)	0 (0.00%)	
Molecular Classification				0.128
Luminal	485 (68.50%)	422 (69.98%)	63 (60.00%)	
HER2 positive	90 (12.71%)	75 (12.44%)	15 (14.29%)	
Triple negative	130 (18.36%)	103 (17.08%)	27 (25.71%)	
unknown	3 (0.42%)	3 (0.50%)	0 (0.00%)	
radiotherapy				< 0.001
No	439 (62.01%)	395 (65.51%)	44 (41.90%)	
Yes	173 (24.44%)	153 (25.37%)	20 (19.05%)	
unknown	96 (13.56%)	55 (9.12%)	41 (39.05%)	
chemotherapy				< 0.001
No	121 (17.09%)	96 (15.92%)	25 (23.81%)	
Yes	546 (77.12%)	483 (80.10%)	63 (60.00%)	
unknown	41 (5.79%)	24 (3.98%)	17 (16.19%)	

The distribution of patient and tumor characteristics was analyzed in relation to SDC1 staining in tumor and stromal cells. The presence of SDC1 in tumor cells was significantly correlated with TNM stage, molecular classification, and radiotherapy, as indicated in table S2.

**Fig. 7:**
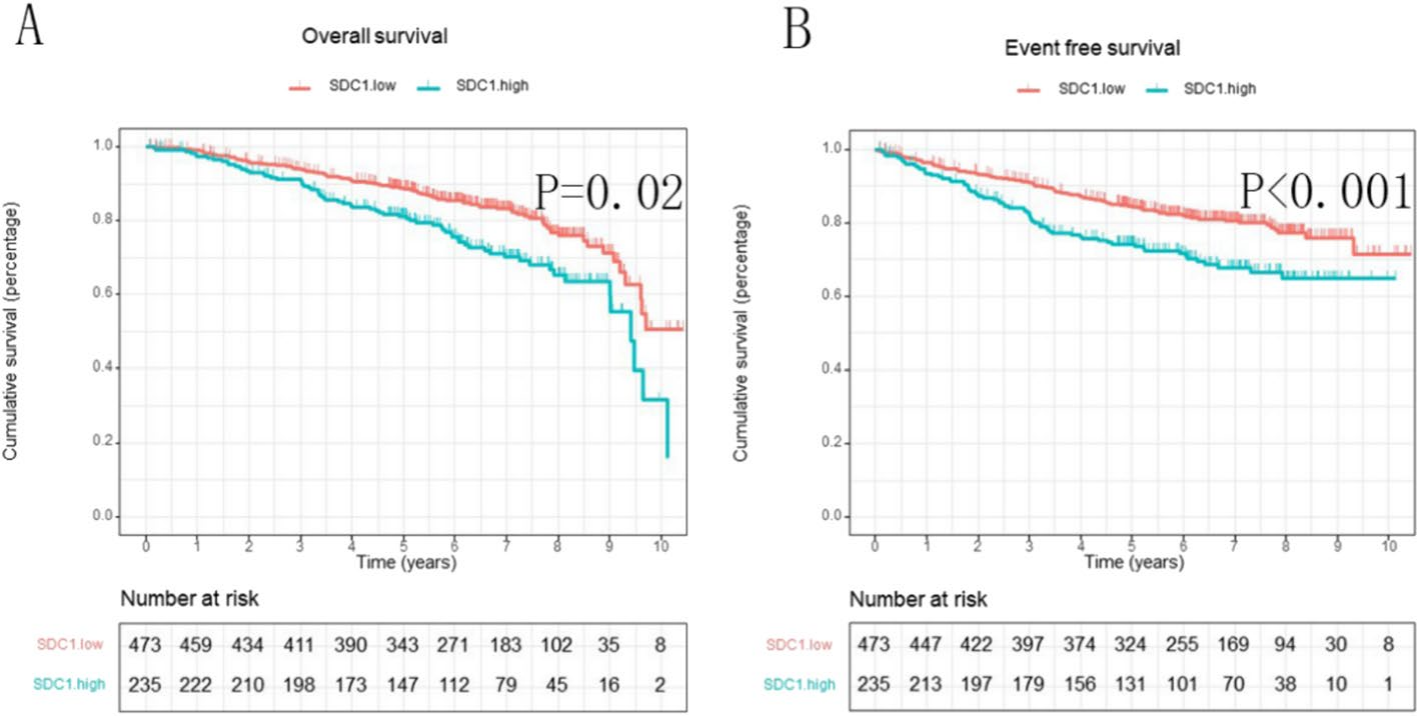
SDC1 expression in tumor cells was significantly associated with patient OS and EFS. **A** The association of SDC1 expression in tumor cells and patients’ OS; **B** The association of SDC1 expression in tumor cells and patients’ EFS

We further assessed the relationship of SDC1 expression (in overall tumor tissue, tumor cells, and stromal cells) with patient OS and EFS using multivariable Cox regression, adjusting for clinical variables including age, tumor characteristics, nodal status, menopausal status, radiotherapy, chemotherapy, vascular invasion, and TNM stage. The results demonstrated that high SDC1 expression in tumor cells was an independent prognostic factor associated with worse OS [HR: 1.54 (95% CI 1.01–2.35), P = 0.044] and EFS [HR: 1.80 (95% CI 1.29–2.53), P = 0.001] (Fig. 7). In contrast, SDC1 expression in tumor tissue and stromal cells did not show a significant association with patient OS and EFS, as shown in Tables 2 and 3, FigS1.

**Table 2:** The relationship between SDC1 expressed in different pattern of tumor tissue and OS in cancer patients

	Crude model		Multivariable adjusted model 1		Multivariable adjusted model 2	
	HR (95% CI)	P value	HR (95% CI)	P value	HR (95% CI)	P value
SDC1 expressed in total tumor tissue						
Low	1.0		1.0		1.0	
high	1.20(0.81,1.78)	0.367	1.23(0.83,1.84)	0.298	1.25(0.82,1.92)	0.299
SDC1 expressed in tumor cell						
Low	1.0		1.0		1.0	
High	1.57 (1.06, 2.31)	0.0235	1.51 (1.02, 2.24)	0.0379	1.54 (1.01, 2.35)	0.0441
SDC1 expressed in stromal cell						
Low	1.0		1.0		1.0	
High	0.72 (0.48, 1.07)	0.1022	0.84 (0.56, 1.26)	0.3958	0.78 (0.51, 1.18)	0.2395

Multivariable Adjusted Model 1 adjust for: age, Tumor and Nodal

Multivariable Adjusted Model 2 adjust for: age, Tumor,Nodal, menstruation, radiotherapy, chemotherapy, Vascular invasion, and TNM stage

**Table 3:** The relationship between SDC1 expressed in different pattern of tumor tissue and EFS in cancer patients

	Crude model		Multivariable adjusted model 1		Multivariable adjusted model 2	
	HR (95% CI)	P value	HR (95% CI)	HR (95% CI)	P value	HR (95% CI)
SDC1 expressed in total tumor tissue						
Low	1.0		1.0		1.0	
high	1.31 (0.95, 1.81)	0.098	1.40 (1.01,1.93)	0.042	1.40 (1.00, 1.96)	0.0510
SDC1 expressed in tumor cell						
Low	1.0		1.0		1.0	
High	1.74 (1.27, 2.39)	0.0006	1.77 (1.29, 2.44)	0.0005	1.80 (1.29, 2.53)	0.0006
SDC1 expressed in stromal cell						
Low	1.0		1.0		1.0	
High	0.78 (0.56, 1.07)	0.1243	0.86 (0.62, 1.20)	0.3757	0.81 (0.58, 1.13)	0.2154

Multivariable Adjusted Model 1 adjust for: age, Tumor and Nodal

Multivariable Adjusted Model 2 adjust for: age, Tumor,Nodal, menstruation, radiotherapy, chemotherapy, Vascular invasion, and TNM stage

### Different pattern of SDC1 expression in relation to breast cancer patients’ OS stratified by molecular subtypes and other factors

We examined the relationship between SDC1 expression (in tumor and stromal cells) and patient OS and event-free survival (EFS) using a multivariable Cox regression model, adjusting for clinically significant variables including age, tumor characteristics, nodal involvement, menstruation status, radiotherapy, chemotherapy, vascular invasion, and TNM stage across different molecular subtypes: Luminal, HER2 + , and triple-negative breast cancer.

In the Luminal subtype, high SDC1 expression in tumor cells was significantly associated with worse patient OS [HR: 1.82 (1.02, 3.25), P = 0.0443], whereas no significant association was observed in HER2 + and triple-negative breast cancer [HR: 0.95 (0.24, 3.71), P = 0.9384 for both]. Conversely, in HER2 + breast cancer, SDC1 expression in stromal cells was significantly associated with improved OS [HR: 0.14 (0.02, 0.85), P = 0.0326], but not found in Luminal or triple-negative subtypes [HR: 1.00 (0.57, 1.73), P = 0.9861 and HR: 0.46 (0.17, 1.30), P = 0.1449, respectively], as detailed in Table 4.

**Table 4:** The relationship between SDC1 expressed in different pattern of tumor tissue and OS in different subtype breast cancer patients

	Crude model		Multivariable adjusted model 1		Multivariable adjusted model 2	
	HR (95% CI)	P value	HR (95% CI)	P value	HR (95% CI)	P value
Luminal subtype SDC1 expressed in tumor cell						
Low	1.0		1.0		1.0	
high	1.67 (0.98, 2.84)	0.0588	1.63 (0.95, 2.82)	0.0775	1.82 (1.02, 3.25)	0.0443
SDC1 expressed in stromal cell						
Low	1.0		1.0		1.0	
High	0.90 (0.54, 1.50)	0.6823	1.03 (0.61, 1.74)	0.8997	1.00 (0.57, 1.73)	0.9861
HER2 + subtype						
SDC1 expressed in tumor cell						
Low	1.0		1.0		1.0	
high	0.78 (0.28, 2.15)	0.6310	0.74 (0.24, 2.24)	0.5923	0.95 (0.24, 3.71)	0.9384
SDC1 expressed in stromal cell						
Low	1.0		1.0		1.0	
High	0.48 (0.16, 1.41)	0.1805	0.30 (0.09, 1.04)	0.0573	0.14 (0.02, 0.85)	0.0326
Triple negative subtype						
SDC1 expressed in tumor cell						
Low	1.0		1.0		1.0	
high	1.46 (0.68, 3.15)	0.3333	1.00 (0.43, 2.32)	0.9943	0.97 (0.37, 2.54)	0.9435
SDC1 expressed in stromal cell						
Low	1.0		1.0		1.0	
High	0.90 (0.54, 1.50)	0.6823	1.03 (0.61, 1.74)	0.8997	1.00 (0.57, 1.73)	0.9861

Multivariable Adjusted Model 1 adjust for: age, Tumor and Nodal

Multivariable Adjusted Model 2 adjust for: age, Tumor,Nodal, menstruation, radiotherapy, chemotherapy, Vascular invasion, and TNM stage

**Fig. 8:**
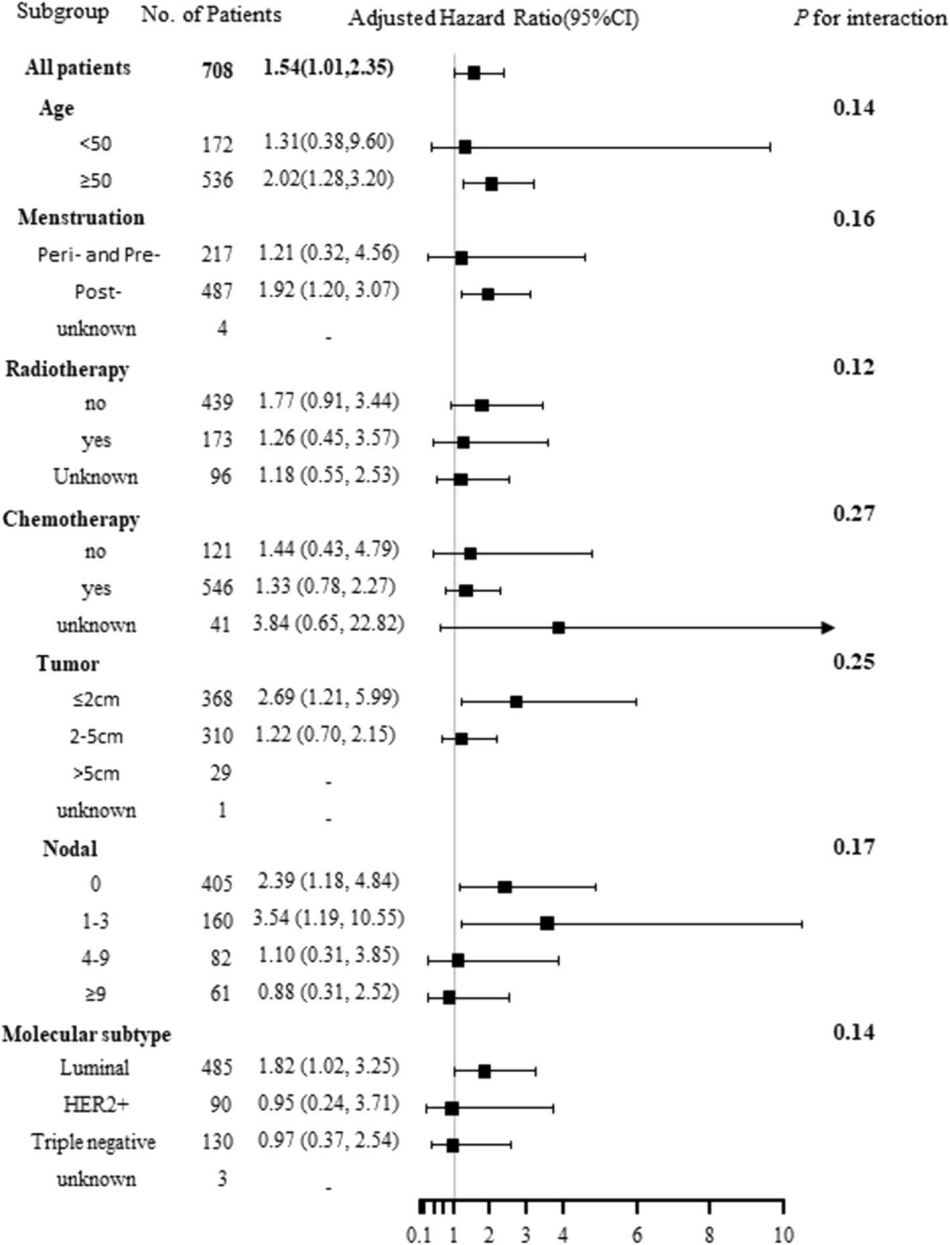
The association of SDC1 expression in tumor cells and patents’ OS in subtype analysis

Subgroup analysis of clinical factors further revealed that the association between SDC1 expression in tumor cells and OS was particularly evident in patients aged 50 years or older, post-menopausal women, tumors ≤ 2 cm, and those with 0–3 nodal involvement. In contrast, this association was not observed in patients younger than 50, pre- or peri-menopausal women, tumors > 2 cm, and those with more than 3 nodal involvement, as illustrated in Fig. 8.

### The cell proliferation, colony formation and stem cell marker expression were reduced after knocking down of SDC1 expression in breast cancer cell

**Fig. 9:**
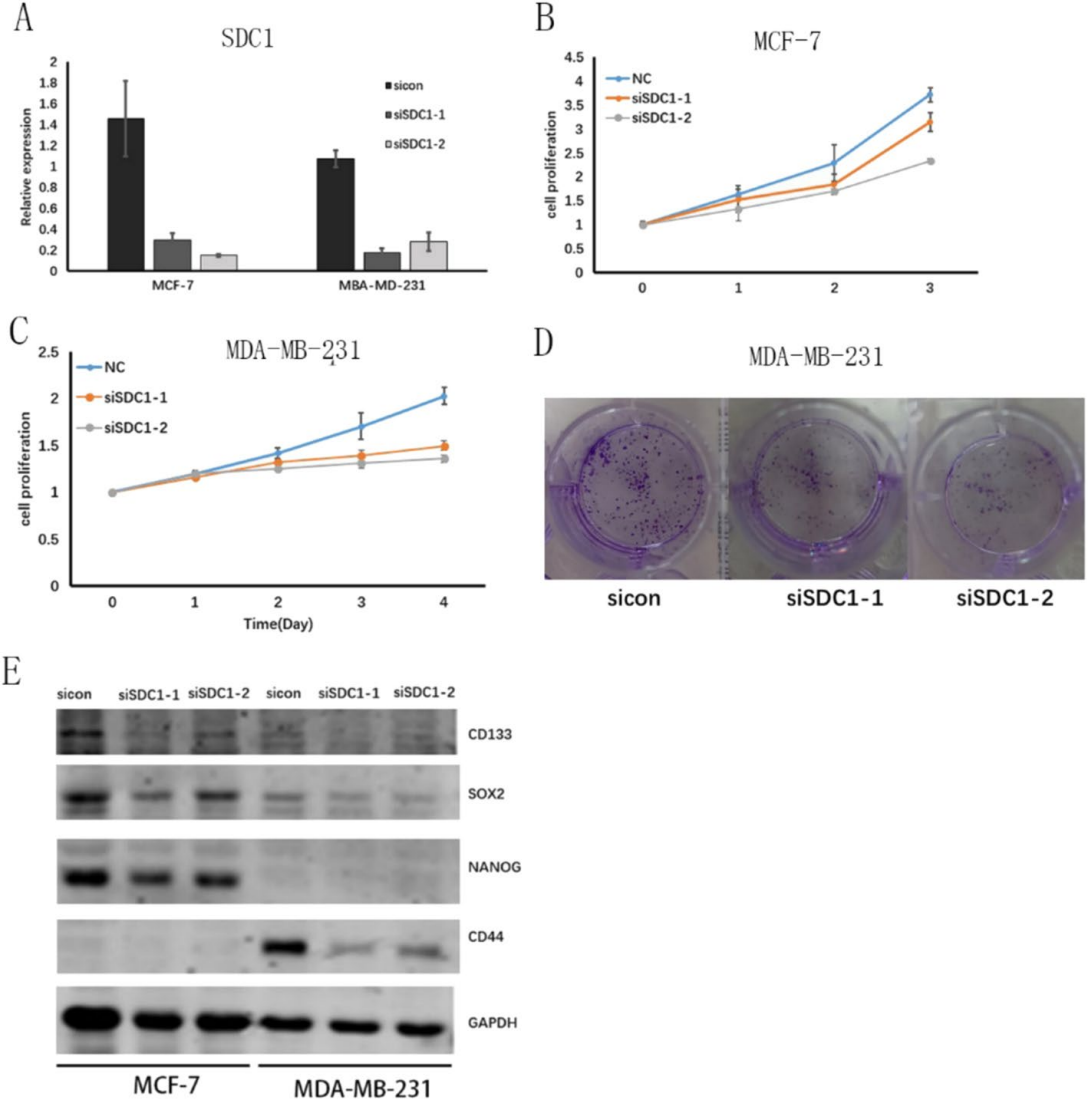
The cell proliferation and stem cell marker expression after knocking down of SDC1 expression in breast cancer cell. **A** SDC1 expression after knocking down of SDC1 expression in breast cancer cell; **B** MCF-7 cell proliferation after knocking down SDC1 expression. **C** MDA-MB-231 cell proliferation after knocking down SDC1 expression. **D** MDA-MB-231 colony formation after knocking down SDC1 expression. **E** The stem cell markers expression after knocking down of SDC1 in breast cancer cell by Western blot

Transfection of MDA-MB-231 and MCF-7 cells with SDC1-targeting siRNAs (siSDC1-1 and siSDC1-2) significantly reduced SDC1 expression, as confirmed by qPCR (Fig. 9A). This knockdown of SDC1 subsequently led to a marked inhibition of cell proliferation in both cell lines (Fig. 9B, C) and inhibited colony formation in MDA-MB-231 cells (Fig. 9D). In contrast, the addition of a CD133 antibody to the culture medium did not alter the proliferation of either MDA-MB-231 or MCF-7 cells (Fig. S2A, B).

Western blot analysis was performed to evaluate the protein levels of stem cell biomarkers (CD133, SOX2, NANOG, and CD44) following SDC1 knockdown. In MDA-MB-231 cells, SDC1 silencing significantly reduced the expression of CD44 and SOX2, whereas in MCF-7 cells, it led to a notable decrease in CD133, SOX2, and NANOG. However, NANOG and CD133 could not be detected in MDA-MB-231 cells, and CD44 was absent in MCF-7 cells, as shown in (Fig. 9E, FigS3).

Furthermore, a multivariable Cox regression model, adjusted for clinically relevant factors (including age, tumor characteristics, nodal involvement, menstruation, radiotherapy, chemotherapy, vascular invasion, and TNM stage) revealed a significant association between tumor cell SDC1 expression and CD24 expression [HR:1.98 (95% CI 1.38–2.84), P = 0.0002], as presented in Table 5.

**Table 5:** SDC1 expressed in tumor cell positive correlated with CD24 expressed in tumor tissue

The correlation of SDC1 and CD24 expression	Non-adjusted HR (95% CI), P value	Adjust I HR (95% CI), P value	Adjust II HR (95% CI), P value
Low	1.0	1.0	1.0
High	2.41 (1.71, 3.38) < 0.0001	2.34 (1.66,3.30) < 0.0001	1.98 (1.38, 2.84) 0.0002

Multivariable Adjusted Model 1 adjust for: age, Tumor and Nodal

Multivariable Adjusted Model 2 adjust for: age, Tumor, Nodal, menstruation, radiotherapy, chemotherapy, Vascular invasion, and TNM stage

## Discussion

Although new diagnostic and target therapy technologies have been developed for cancer therapy, their efficacy in advanced cancer therapy remains poor. It is very important to understand the molecular mechanisms underlying cancer development. Previously, we used the TCGA dataset to determine that SDC1 might be a prognostic marker in breast cancer through immune cell infiltration. Several studies have indicated that SDC1 plays an important role in the development of colon, pancreatic, breast and liver cancers, few studies on SDC1 in pan-cancer have been published [6–9].

In our study, we found that SDC1 was significantly associated with poor OS in BRCA, GBM, LGG, MESO and PAAD. SDC1 expression was associated with immune cell infiltration in almost all cancers by TCGA dataset, though this requires further validation. Liu et.al. indicated that Loss of SDC1 enhances the sensitivity of tumor cells to CD8 + T cell cytotoxicity in B16F10 cells [17]. Chen indicated that T cells in PDAC have enriched expression of CCL5 which could induce tumor cell migration through interaction with SDC1[18]. In tumor stemness analysis, we found that SDC1 was positively correlated with the scores for DNA, EREG-methss, DMPss, ENHss, RNAss and EREG in STES, THTM, but SDC1 expression was negatively correlated with the scores for DNA, EREG-methss, DMPss, ENHss, RNAss and EREG in TGCT, LUAD in TCGA dataset. SDC1 relies on the IL-6/STAT3, Notch and EGFR pathways to induce stem cell formation in breast cancer, Ibrahim et.al. indicated that SDC1 can modulate the triple negative inflammatory breast cancer stem cell phenotype via the IL-6/STAT3, Notch and EGFR signaling pathways [19]. However, further exploration in vitro and vivo is needed for other cancer types.

Furthermore, we focused on breast cancer. We discovered that the expression of SDC1 in tumor cells was positively correlated with overall survival (OS) and event-free survival (EFS) in breast cancer patients, particularly among those with luminal subtypes. However, this association was not observed in HER2 + positive or triple-negative breast cancer patients when analyzed using a multivariable Cox regression model that included relevant patient, tumor, and treatment factors. Conversely, SDC1 expression in stromal cells did not show any correlation with OS and EFS in breast cancer patients. Simon et al. noted that different expression patterns of SDC1 could indicate varying prognostic implications. Specifically, cytoplasmic SDC1 expression in breast cancer was associated with more aggressive behavior, while stromal SDC1 expression correlated with a better prognosis according to Kaplan–Meier survival analysis, although this was not supported by multivariate analyses [20]. The presence of SDC1 or CD138 in tumor cells could provide prognostic insights when combined with clinical parameters. The differing results may be attributed to the molecular subtypes of the patients analyzed. For luminal breast cancer patients, tumor cell SDC1 expression was positively associated with OS and EFS, but this was not the case for HER2 + and triple-negative patients. In our data, nearly two-thirds of the patients were of the luminal subtype, with luminal patients making up 68.5%, compared to 12.7% for HER2 + patients and 18.4% for triple-negative patients. The negative association of stromal SDC1 with HER2 + breast cancer suggests its significant role in immune therapy through TIL cell infiltration. TIL cells play a beneficial role in HER2 positive and triple-negative breast cancers, but not in luminal subtypes [21–23]. Zhong found that low SDC1 expression in CAFs in triple-negative breast cancer was linked to poor survival due to TIL cell infiltration [24]. However, in luminal breast cancer, immune activity had a less favorable impact on tumor progression, with CD8 + T cell infiltration more likely leading to invasion. In Luminal B subtype breast cancer, sTIL cell infiltration was associated with a shorter relapse-free interval [25, 26].

In the analysis of clinical subtypes, findings revealed that in older patients, particularly those aged 50 and above and post-menopausal, the expression of SDC1 in tumor cells was significantly linked to overall survival (OS), unlike in younger patients. In postmenopausal women, hormonal levels can be disrupted. Various studies have shown a close relationship between SDC1 expression and steroid levels [27–30]. Hofling et al. examined the histopathological expression of SDC1 in breast tissue from cynomolgus monkeys undergoing long-term hormonal treatment with three different androgens, including tibolone (a selective synthetic steroid), conjugated equine estrogens (CEE), and medroxyprogesterone acetate (MPA). They found that SDC1 expression significantly increased in stromal tissue with tibolone and the combination of CEE/MPA, while CEE alone did not have a significant impact [31]. Additionally, SDC1 expression levels were influenced by omega-3 polyunsaturated fatty acids (n-3 PUFA) in human breast cancer cells, with n-3 PUFA-enriched low-density lipoprotein (LDL) affecting SDC1-mediated biological processes [27]. In terms of stage analysis, SDC1 expression in tumor cells was associated with OS in patients with T1-2 and N0-1 stages, but not in those with advanced breast cancer, suggesting that SDC1 plays a crucial role in the early progression of breast cancer. In pancreatic cancer, serum SDC1 has prognostic value in early-stage patients but not in advanced cases. Similarly, in human colorectal cancer, serum SDC1 levels serve as a prognostic biomarker for stages II and III, but not for stage IV [31, 32]. This suggests that early breast cancer patients with positive SDC1 staining in tumor cells may require intensified chemotherapy or radiotherapy.

Moreover, we explored the mechanism behind the significant role of SDC1 expressed in tumor cells during tumor progression. Silencing SDC1 in breast cancer cells inhibited their proliferation and reduced the protein levels of stem markers CD44, CD133, and SOX2. Additionally, analysis of the TCGA dataset showed a strong correlation between SDC1 expression and cancer stem markers. In microarray tissue studies, SDC1 was positively correlated with the stem marker CD24. Previous research has confirmed that SDC1 is crucial for the formation of cancer stem cells in colon cancer, where its depletion was found to enhance colon cancer invasion and cancer stem cell formation by activating integrins and FAK. SDC1 also plays a role in maintaining the osteo-adipogenic balance of human mesenchymal stem cells [33, 34], but further experiment needs to be conducted to verify that SDC1 can induce cancer cell proliferation through stem cell formation. However, adding CD138 antibody to the cell medium did not affect cell proliferation. Several studies have indicated that soluble SDC1 is important for tumor cell progression [35–37], which contradicts our findings for two reasons: first, in vitro studies lack the tumor microenvironment, and second, SDC1 expressed in stromal cells is associated with immune cells.

In our research, we found that SDC1 expression in tumor cells is vital for tumor progression, particularly in Luminal, older, and early-stage breast cancer patients. Targeting SDC1 expression in tumor cells could be a potential therapeutic approach for breast cancer. However, further in vivo studies are needed to determine the significance of neutralizing sSDC1 in breast cancer treatment.

## Limitations

There are some limitations in our study. First, while the pan-cancer analysis of SDC1 was conducted using the TCGA dataset, functional validation through in vivo or in vitro experiments was performed only in breast cancer, leaving its precise role in other cancer types unverified. Second, the observed correlations between SDC1 expression and immune cell infiltration or cancer stemness remain preliminary; more mechanistic studies are needed to definitively establish the causal relationships in these processes.

## Conclusions

Our study revealed that SDC1 plays diverse roles in pan-cancer, which may be correlated with immune cell infiltration and cell stemness based on the TCGA dataset. Furthermore, we verified the result in breast cancer cells, indicating that higher SDC1 expression correlates with increased patient mortality, especially in the Luminal molecular subtype. Therefore, elevated SDC1 levels in tumor cells might serve as a negative prognostic indicator for breast cancer and a potential therapeutic target, particularly in the Luminal subtype.

## Supplementary Information

Additional file 1. Figure S1: SDC1 expression in tumor tissue and stromal cells was not significantly associated with patient OS. **A** The association of SDC1 expression in tumor tissue and patients’ OS; **B** The association of SDC1 expression in stromal cells and patients’ OS. Figure S2: The cell proliferation after adding CD138 antibody to the medium in MB-MDA231 (**A**) and MCF-7 (**B**)

Additional file 2.

## Data Availability

The RNA expression data from TCGA were obtained directly from the TCGA database (https://portal.gdc.cancer.gov), GSE176078 (PMID: 34493872), GSE161529 (PMID: 33950524), and GSE148673 (PMID: 33462507) analyzed for SDC1 expression clusters can be checked in TISCH2 (http://tisch.comp-genomics.org/). For more information about the datasets used in this study, please contact the corresponding author.
