# Supplementary material for: Investigation of the pan-cancer property of SDC1 and its expression pattern affected patients’ overall survival for breast cancer
Source: Discov Oncol. 2025 Dec 9;16:2197. doi: 10.1007/s12672-025-04139-x (PMC12705517; doi:10.1007/s12672-025-04139-x)
Supplement: Supplementary file 1 — Additional file 1. Figure S1: SDC1 expression in tumor tissue and stromal cells was not significantly associated with patient OS. A The association of SDC1 expression in tumor tissue and patients’ OS; B The association of SDC1 expression in stromal cells and patients’ OS. Figure S2: The cell proliferation after adding CD138 antibody to the medium in MB-MDA231 (A) and MCF-7 (B) [file 12672_2025_4139_MOESM1_ESM.docx]

Table S1 Description of the clinical characters and EFS

| EFS | No(552) | Yes(156) | P-value |
| --- | --- | --- | --- |
| Age(average±SD) | 57.05 ± 11.94 | 61.14 ± 13.43 | <0.001 |
| Age |  |  | 0.060 |
| <50 | 143 (25.91%) | 29 (18.59%) |  |
| ≥50 | 409 (74.09%) | 127 (81.41%) |  |
| menstruation |  |  | 0.012 |
| Peri- and Pre- | 183 (33.15%) | 34 (21.79%) |  |
| Post- | 365 (66.12%) | 122 (78.21%) |  |
| unknown | 4 (0.72%) | 0 (0.00%) |  |
| surgical method |  |  | 0.648 |
| conservation | 44 (7.97%) | 13 (8.33%) |  |
| radical | 505 (91.49%) | 143 (91.67%) |  |
| unknown | 3 (0.54%) | 0 (0.00%) |  |
| Tumor |  |  | 0.016 |
| ≤2cm | 302 (54.71%) | 66 (42.31%) |  |
| 2-5cm | 231 (41.85%) | 79 (50.64%) |  |
| >5cm | 18 (3.26%) | 11 (7.05%) |  |
| unknown | 1 (0.18%) | 0 (0.00%) |  |
| Nodal |  |  | <0.001 |
| 0 | 338 (61.23%) | 67 (42.95%) |  |
| 1-3 | 127 (23.01%) | 33 (21.15%) |  |
| 4-9 | 58 (10.51%) | 24 (15.38%) |  |
| ≥9 | 29 (5.25%) | 32 (20.51%) |  |
| TNM stage |  |  | <0.001 |
| I | 210 (38.04%) | 38 (24.36%) |  |
| IIA | 220 (39.86%) | 49 (31.41%) |  |
| IIB-IIIA | 87 (15.76%) | 35 (22.44%) |  |
| IIIB-IIIC | 30 (5.43%) | 34 (21.79%) |  |
| unknown | 5 (0.91%) | 0 (0.00%) |  |
| Grade level |  |  | 0.157 |
| 1 | 15 (2.72%) | 0 (0.00%) |  |
| 2 | 263 (47.64%) | 74 (47.44%) |  |
| 3 | 266 (48.19%) | 81 (51.92%) |  |
| unknown | 8 (1.45%) | 1 (0.64%) |  |
| Vascular invasion |  |  | <0.001 |
| no | 466 (84.42%) | 107 (68.59%) |  |
| yes | 80 (14.49%) | 48 (30.77%) |  |
| unknown | 6 (1.09%) | 1 (0.64%) |  |
| Resection margin |  |  | 0.941 |
| negative | 544 (98.55%) | 154 (98.72%) |  |
| positive | 3 (0.54%) | 1 (0.64%) |  |
| unknown | 5 (0.91%) | 1 (0.64%) |  |
| ER (estrogen receptor) |  |  | 0.138 |
| negative | 170 (30.80%) | 60 (38.46%) |  |
| positive | 379 (68.66%) | 96 (61.54%) |  |
| unknown | 3 (0.54%) | 0 (0.00%) |  |
| PR(Progenstogen receptor) |  |  | 0.089 |
| negative | 222 (40.22%) | 78 (50.00%) |  |
| positive | 327 (59.24%) | 77 (49.36%) |  |
| unknown | 3 (0.54%) | 1 (0.64%) |  |
| HER2(FISH) |  |  | 0.497 |
| negative | 432 (78.26%) | 120 (76.92%) |  |
| positive | 116 (21.01%) | 36 (23.08%) |  |
| unknown | 4 (0.72%) | 0 (0.00%) |  |
| Molecular Classification |  |  | 0.236 |
| Luminal | 387 (70.11%) | 98 (62.82%) |  |
| HER2 positive | 67 (12.14%) | 23 (14.74%) |  |
| Triple negative | 95 (17.21%) | 35 (22.44%) |  |
| unknown | 3 (0.54%) | 0 (0.00%) |  |
| radiotherapy |  |  | <0.001 |
| No | 370 (67.03%) | 69 (44.23%) |  |
| Yes | 138 (25.00%) | 35 (22.44%) |  |
| unknown | 44 (7.97%) | 52 (33.33%) |  |
| chemotherapy |  |  | 0.003 |
| No | 90 (16.30%) | 31 (19.87%) |  |
| Yes | 438 (79.35%) | 108 (69.23%) |  |
| unknown | 24 (4.35%) | 17 (10.90%) |  |

Table S2 Distribution of tumor cell SDC1 staining and clinical characters.

| **SDC1 expression in**  **tumor cell** | **low(473)** | **high(235)** | **P-value** |
| --- | --- | --- | --- |
| Age(average±SD) | 58.07 ± 12.62 | 57.70 ± 11.93 | 0.706 |
| Age |  |  | 0.257 |
| <50 | 121 (25.58%) | 51 (21.70%) |  |
| ≥50 | 352 (74.42%) | 184 (78.30%) |  |
| menstruation |  |  | 0.186 |
| Peri- and Pre- | 148 (31.29%) | 69 (29.36%) |  |
| Post- | 324 (68.50%) | 163 (69.36%) |  |
| unknown | 1 (0.21%) | 3 (1.28%) |  |
| Tumor |  |  | 0.084 |
| ≤2cm | 261 (55.18%) | 107 (45.53%) |  |
| 2-5cm | 192 (40.59%) | 118 (50.21%) |  |
| >5cm | 19 (4.02%) | 10 (4.26%) |  |
| unknown | 1 (0.21%) | 0 (0.00%) |  |
| Nodal |  |  | 0.904 |
| 0 | 274 (57.93%) | 131 (55.74%) |  |
| 1-3 | 103 (21.78%) | 57 (24.26%) |  |
| 4-9 | 55 (11.63%) | 27 (11.49%) |  |
| ≥9 | 41 (8.67%) | 20 (8.51%) |  |
| TNM stage |  |  | <0.001 |
| I | 192 (40.59%) | 56 (23.83%) |  |
| IIA | 175 (37.00%) | 94 (40.00%) |  |
| IIB-IIIA | 68 (14.38%) | 54 (22.98%) |  |
| IIIB-IIIC | 36 (7.61%) | 28 (11.91%) |  |
| unknown | 2 (0.42%) | 3 (1.28%) |  |
| Tumor grade |  |  | 0.001 |
| 1 | 13 (2.75%) | 2 (0.85%) |  |
| 2 | 246 (52.01%) | 91 (38.72%) |  |
| 3 | 208 (43.97%) | 139 (59.15%) |  |
| 4 | 6 (1.27%) | 3 (1.28%) |  |
| Molecular Classification |  |  | <0.001 |
| Luminal | 372 (78.65%) | 113 (48.09%) |  |
| HER2 positive | 36 (7.61%) | 54 (22.98%) |  |
| Triple negative | 64 (13.53%) | 66 (28.09%) |  |
| unknown | 1 (0.21%) | 2 (0.85%) |  |
| radiotherapy |  |  | 0.049 |
| No | 297 (62.79%) | 142 (60.43%) |  |
| Yes | 122 (25.79%) | 51 (21.70%) |  |
| unknown | 54 (11.42%) | 42 (17.87%) |  |
| chemotherapy |  |  | 0.822 |
| No | 83 (17.55%) | 38 (16.17%) |  |
| Yes | 364 (76.96%) | 182 (77.45%) |  |
| unknown | 26 (5.50%) | 15 (6.38%) |  |
