# Supplementary figures and images for: Investigation of the pan-cancer property of SDC1 and its expression pattern affected patients’ overall survival for breast cancer
Source: Discov Oncol. 2025 Dec 9;16:2197. doi: 10.1007/s12672-025-04139-x (PMC12705517; doi:10.1007/s12672-025-04139-x)

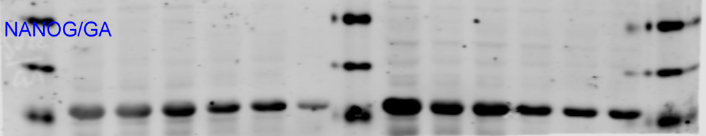

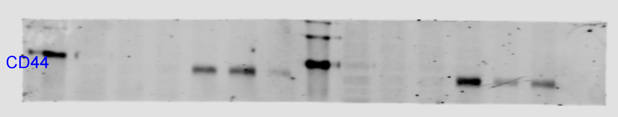

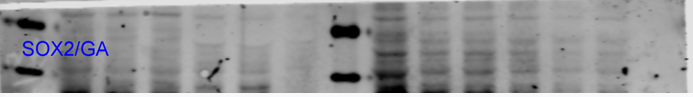

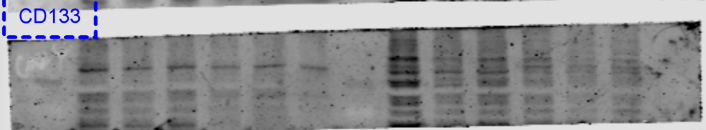

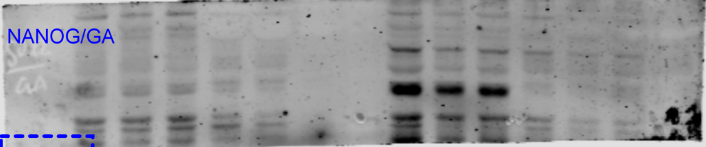
Figure S3

sicon

siSDC1-2

siSDC1-1

siSDC1-2

siSDC1-1

GAPDH

CD44

SOX2

CD133

NANOG

**MCF-7**

**MDA-MB-231**

sicon

Supplement: Supplementary file 2 — Additional file 2. [file 12672_2025_4139_MOESM2_ESM.docx]
